# Passive targeting and lung tolerability of enoxaparin microspheres for a sustained antithrombotic activity in rats

**DOI:** 10.1080/10717544.2016.1245368

**Published:** 2017-02-03

**Authors:** Shaimaa S. Ibrahim, Rihab Osman, Nahed D. Mortada, Ahmed-Shawky Geneidy, Gehanne A. S. Awad

**Affiliations:** Department of Pharmaceutics and Industrial Pharmacy, Faculty of Pharmacy, Ain Shams University, Cairo, Egypt

**Keywords:** Albumin, enoxaparin, microspheres, passive lung targeting, controlled delivery, anticoagulant

## Abstract

Pulmonary bed can retain microparticles (MP) larger than their capillaries’ diameter, hence we offer a promising way for lung passive targeting following intravenous (IV) administration. In this study, enoxaparin (Enox)-albumin microspheres (Enox-Alb MS) were, optimally, developed as lung targeted sustained release MP for IV use. Lung tolerability and targeting efficiency of Enox-Alb MS were tested, and the pharmacokinetic profile following IV administration to albino rats was constructed. *In vivo* studies confirmed high lung targeting efficiency of Enox-Alb MS with lack of potential tissue toxicity. The anticoagulant activity of the selected Alb MS was significantly sustained for up to 38 h compared to 5 h for the market product. Alb MS are promising delivery carriers for controlled and targeted delivery of Enox to the lungs for prophylaxis and treatment of pulmonary embolism.

## Introduction

Pulmonary embolism (PE), the main life threatening complication of venous thrombo-embolism (VTE), constitutes the third most common cause of vascular death after myocardial infarction and stroke, and the leading preventable cause of death in hospitalized patients (Walter et al., 2014). Prophylaxis and treatment of PE are achieved by anticoagulation therapies including low molecular weight heparins (LMWHs) (Bai & Ahsan, 2010).

LMWHs are water soluble, highly sulfated, depolymerized heparin fragments that inhibit blood clotting by binding to antithrombin, thereby catalyzing the inactivation of factorXa. This inhibits thrombin and suppresses the cascade of reactions that lead to blood clotting (Moon et al., 2006). In addition to their widespread use in the treatment and prophylaxis of VTE, LMWHs have also been used for maintenance of vessel patency during hemodialysis and artery bypass grafting, prevention of acute bronchoconstrictor responses and airway hyper-responsiveness in asthma and regulation of growth factor activity in various vascular disorders and angiogenesis (Ibrahim et al., 2015). Among the LMWHs, enoxaparin (Enox) has the broadest range of FDA-approved indications, and thus is the most widely used, as assessed by studies of clinician’s practices and clinical care (Merli & Groce, 2010). Enox parenteral solution, Clexane®, is the only available dosage form in the market, as the drug’s physico-chemical properties hinder its absorption from the GIT and most other mucosal surfaces (Baldwin et al., 2012). Unfortunately, Clexane® suffers from short half-life (4.5 h) and general distribution of the drug to all body parts, resulting in the need for daily parenteral administration of large doses with increased risk of hemorrhage especially with long-term LMWH usage. Hence, it is desirable to modify the current treatment approach (Lanke et al., 2009; Bai & Ahsan, 2010).

A number of reports have been published on the biodistribution of injected particles including poly(lactic-co-glycolic acid) (Huo et al., 2005), polystyrene (Chao et al., 2010; Kutscher et al., 2010) and albumin (Willmott et al., 1985; Sahin et al., 2002), for pulmonary drug delivery following IV administration. Albumin microspheres (Alb MS) have been extensively investigated for drug targeting and controlled delivery of therapeutic agents to plasma, cells or organs (Thakkar et al., 2005). The exploitable features of Alb include: biodegradation, biocompatibility, ability to interact with a wide variety of drugs and non-antigenicity. In addition, it exhibits better stability, shelf life and higher loading capacity for hydrophilic molecules compared to other colloidal carriers such as liposomes (Müller et al., 1996; Thakkar et al., 2005; Iemma et al., 2009).

Lung targeting and retention properties of injectable microparticles (MP) rely on the mechanical filter property of pulmonary capillary bed entrapping them, following IV administration. Microemboli are formed by embedding the particles in the capillaries, not the arterioles. After blockage, the lungs continue to function normally, due to the unused capillaries recruitment. The developed lung-targeted MP should maintain normal microvascular hemodynamics of the pulmonary circulation to avoid potential toxicity caused by vascular occlusion in acute massive embolism. Accordingly particle size (PS) engineering is a key property for lung distribution by targeting the capillary bed of the lungs while allowing unhindered blood flow (Gupta & Hung, 1989; Kutscher et al., 2010; Deshmukh et al., 2012).

For monitoring bioactivity of Enox, assay of activated partial thromboplastin time (aPTT) (Sun et al., 2010) or chromogenic measurement of its antifactorXa activity in blood samples (Rawat et al., 2008a) has been reported. However, aPTT assay offers a relatively insensitive measure of LMWHs in general due to their low inhibitory activity against thrombin compared to factorXa (Lee et al., 2001). Furthermore, assay of antifactorXa activity is the currently recommended method for LMWH therapy monitoring (Priglinger et al., 2003). Plasma antifactorXa levels were assumed to be a reflection of the drug activity levels in the lungs.

To date, a drug delivery system for passive targeting of LMWHs to lungs for treatment and prophylaxis of PE, has not been investigated. Hence, the current work was designed to study the acute toxicity, efficacy, lung retention and pharmacokinetics (PK) profile of formulated Enox-albumin microspheres (Enox-Alb MS). Study of formulation parameters for PS optimization was also done.

## Materials and methods

### Materials

Enoxaparin Sodium powder (Enox) was kindly provided by Egyptian Company for Pharmaceutical and Chemical Industries, Cairo, Egypt. Egg albumin (Alb) was purchased from LOBA Chemie, Mumbai, India. Glutaraldehyde was purchased from Fluka Chemika, Neu-Ulm, Germany. Fluorescein isothiocyanate (FITC)-dextran (Mw 4400), Azure II and Sorbitan mono-oleate (span 80) were purchased from Sigma-Aldrich, St. Louis, MO. Egg lecithin (EL), about 90% phosphatidyl choline content, was purchased from Fisher Chemical, Loughborough, UK, light liquid paraffin, potassium dihydrogen phosphate, disodium hydrogen phosphate, sodium chloride, n-hexane were purchased from El-Nasr Pharmaceutical Chemicals, Cairo, Egypt. Dextrose in water (5% w/v) intravenous (IV) infusion B.P. 2001 (D5W) was purchased from Egypt Otsuka Pharmaceutical Company, Cairo, Egypt. Marketed enoxaparin sodium solution for injection (Clexane® 80 mg) was purchased from Sanofi-Aventis, Surrey, UK.

### Methods

#### Method of preparation

*Size optimization of Alb MS*. An emulsion crosslinking method was adopted as previously reported with slight modification (Mathew et al., 2007). In this method, the aqueous phase consisted of 15% w/v Alb solution in distilled water. Table 1 summarizes the various variables studied and their levels. Dropwise addition of aqueous phase to the oil phase was followed by stirring at 750 rpm at room temperature for 10 min and glutaraldehyde addition to initiate crosslinking. The system was left under constant magnetic stirring for 24 h. Microspheres separation was done by centrifugation (7000 rpm for 30 min) then washed three times with n-hexane (5 mL each time) to ensure complete removal of excess oil and glutaraldehyde. A 24 h freeze-drying was applied after sediment suspension in 2 mL water.

**Table 1.** Composition of the prepared plain and drug-loaded Alb MS.

Plain albumin microspheres							
	External phase		Surfactant				
Phase volume ratio	Type		Type	Conc. (% w/v)			
0.02	Liquid paraffin		Span 80	−	10		
0.04	Liquid paraffin		Span 80	1	10		
0.04	Olive oil		Span 80	1	–		
0.04	Liquid paraffin		Lecithin	1	5		
Enoxaparin-loaded albumin microspheres							
Surfactant							
Type	Conc. (% w/v)	Theoretical drug loading (% w/w)	Glutaraldehyde conc. (% w/v)			Formula code	
Span 80	1	25	–	2	–	F1	
	2.5	25	–	2	–	F2	
	5	25	–	2	–	F3	
Lecithin	0.5	25	–	2	–	F4	
	1	15	–	2	–	F5	
		20	–	2	–	F6	
		25	1	2	3	F7 F8 F9	
		30	–	2	–	F10	
	2	25	–	2	–	F11	

*Enoxaparin-loading in Alb MS*. Enox was added to the aqueous phase before mixing with the oil phase and then preparation completed as detailed above. Synchronization of PS tuning with drug loading was done by fine modulation of span 80 and lecithin concentrations, different drug loadings and glutaraldehyde concentrations. Compositions of Enox-Alb MS are also shown in Table 1S.

#### Characterization of Alb MS

*Particle size determination*. PS and size distribution were determined by light scattering based on laser diffraction using a Malvern Mastersizer (MasterSizerX, Malvern Instruments Ltd., Malvern, UK). Approximately 2 mg of each powder was dispersed in n-hexane and sonicated for 1 min to disperse any possible agglomerate before measurement (Mathew et al., 2007).

*Estimation of entrapment efficiency % (EE%)*. Evaluation of EE% was a direct estimation of the drug actually present in the MS. Triplicate of accurately weighed 10 mg of lyophilized plain and Enox-loaded MS were suspended in a vial containing 3 mL phosphate buffered saline (PBS) pH 7.4, incubated at 37 °C and agitated at 100 rpm. After 12 days, 0.5 mL sample was carefully separated taking care to avoid collecting any MS, centrifuged at 5000 rpm for 10 min and supernatant assayed for drug concentration by Azure II colorimetric method (Jiao et al., 2002; Rawat et al., 2008a). Absorbance of the respective drug-free formula was used as blank to avoid any interference by formula ingredients, if ever present.

The calculated amount of entrapped drug was considered as the initial drug loading and was used in the release study to calculate percentage of drug (Boonsongrit et al., 2006; Bagre et al., 2013; Jana et al., 2013,2014).

$$\mathrm{EE}\;\%\;=\;\frac{\mathrm{Actual}\;\mathrm{amount}\;\mathrm{of}\;\mathrm{drug}\;\mathrm{in}\;\mathrm{MS}}{\mathrm{Theoretical}\;\mathrm{amount}\;\mathrm{of}\;\mathrm{drug}\;\mathrm{in}\;\mathrm{MS}}\;\times \;100$$

In vitro *Enox release study. In vitro* release study was done using a membrane-less model (Rawat et al., 2008b; Das et al., 2011). Ten milligrams of the selected drug loaded and plain counterpart formulae were accurately weighed and suspended in 3 mL PBS, pH 7.4. The suspensions were incubated at 37 °C in a shaking water bath at 100 strokes/min. At specified time intervals, 0.5 mL samples were carefully withdrawn and replaced with fresh PBS. Samples were centrifuged at 5000 rpm for 10 min. Amount of Enox released was assayed by Azure II colorimetric method.

*Particle behavior in PBS for 48 h*. Fluorescent MS were prepared by replacing the drug with 10 mg of a fluorescent marker FITC-dextran (excitation/emission maxima 490/520 nm) and were incubated in PBS (pH 7.4). At different time intervals up to 48 h, the MS were collected and viewed under an inverted fluorescence microscope using the blue-green channel.

*Scanning electron microscopy (SEM)*. SEM was used to examine shape and surface characteristics of Enox-loaded Alb MS and drug free particles. MS were mounted on an adhesive stub which was then coated with conductive gold with a sputter coater attached to the instrument. Photomicrographs were captured at a voltage accelerator of 20 kV (Mathew et al., 2007; Gülsu et al., 2012).

*Differential scanning calorimetry (DSC)*. DSC was carried out on Enox, Alb, selected Enox-Alb MS and its corresponding drug-free MS. Samples were accurately weighed (3–5 mg) and sealed in aluminum pans with lids. DSC runs were conducted at a rate of 10 °C/min over a temperature range 25–400 °C under dry nitrogen flow rate of 30 mL/min (Das et al., 2011).

*Fourier transform infrared (FT-IR) spectroscopy*. KBr disc method was used for recording FT-IR spectra of Enox, Alb, selected Enox-Alb MS and its corresponding drug-free MS. Pellets of sample and KBr powder were pressed at 20 psi for 5 min using a hydrostatic press. Spectra were then scanned in the wave number range of 4000–400 cm^−1^ at ambient temperature and a resolution of 4 cm^−1^. All spectra were carried under vacuum to remove air humidity contribution to obtain an adequate signal to noise ratio (Mathew et al., 2009).

#### *In vivo* studies

*Animal handling*. In all *in vivo* studies, male albino rats (160–200 g) were injected into their tail vein by a single 200 μL bolus injection of test formulations suspended in D5W. For all animal studies, the experimental procedures conformed to the Ethics Committee of Faculty of Pharmacy, Ain-Shams University on the use of animals.

*Acute toxicity study*. Lung tissues of rats were histologically examined 24 h following IV administration of drug microspheres in a dose equivalent to 100 IU/kg or D5W (as negative control). Rats were anesthetized then sacrificed and the lungs isolated. Lung processing was done according to the method described by Bancroft et al. (1996). The obtained tissue sections were collected on glass slides, deparaffinized and stained by hematoxylin and eosin stain for examination under light microscope. Slides were examined by a board-certified veterinary pathologist, who was blinded in terms of the type of treatment administered to each rat.

*Passive targeting efficiency of Alb MS*. Particulate entrapment in lung tissues was evaluated in terms of MS lung retention and distribution. For this purpose, fluorescence and confocal laser scanning microscopy were conducted. Enox was replaced with FITC-dextran as described earlier under section “Enoxaparin-loading in Alb MS”. FITC-dextran-loaded Alb MS were administered into rat tail veins. A group of untreated rats were allocated as negative control. Animals were sacrificed and their lungs isolated 0.5, 6, 24 and 48 h post-IV administration of FITC-dextran-labeled Alb MS (*n* = 3/time interval). After freezing at −70 °C, lungs were sectioned using a microtome cooled at −25 °C. The tissue cryosections were examined by fluorescent microscope and a confocal microscope coupled with argon-ion laser. Laser used was 30 mW argon laser (488 nm for green channel). The emission was collected using a band pass filter between 505 and 530 nm.

*Pharmacokinetic study*. The study was conducted to compare plasma drug concentration profile versus market product Clexane® through measurement of their antifactorXa activity. Animals were divided into two groups (*n* = 9/group); group I received Clexane® while group II received Enox-Alb MS. Next, blood samples were collected from a capillary in the retro-orbital plexus at specified time intervals of 10 min and 1, 2, 4, 6, 10, 24 and 48 h. The samples were directly mixed with 50 μL of sodium citrate (3.8% solution) and then the plasma was separated by centrifugation (2500 × *g* at 4 °C for 15 min). The obtained plasma samples were stored at −20 °C until further analysis. LMWH concentration was determined by measuring plasma anti-factorXa levels using a colorimetric assay kit (Kinetichrome^TM^ Heparin Anti-Xa kit; Iduron Ltd., Manchester, UK). The assay was performed according to the protocol described by the manufacturer. The principle of the assay is based on the fact that the anticoagulant effect of Enox is mediated via its ability to bind antithrombin III (AT) to inhibit factorXa clotting activity. Enox, in test samples, forms a complex with an added amount of AT (as recommended by assay kit manufacturer). This complex is then incubated with a known excess of factorXa. The amount of factorXa neutralized by Enox-AT complex is directly proportional to the available amount of Enox in the test sample. The remaining amount of factorXa will be evaluated following the addition of a chromogenic peptide substrate, which will be hydrolyzed liberating paranitroaniline (pNA), a chromophoric yellow group. The reaction is acid-stopped by addition of acetic acid and the color intensity is measured spectrophotometrically at 405 nm. An inverse relationship exists between Enox activity and the color intensity. An elaborating diagram is presented in Figure IS.

*Data analysis*. Plain formulations were prepared along with each formula. All measurements were performed in triplicate and data shown are mean with standard deviation (SD).

Data from *in vivo* studies are presented as mean with standard error (SE). Pharmacokinetic analysis to calculate maximum antifactorXa activity as % of baseline level (*C*_max_), time point of maximum antifactorXa activity (*T*_max_), area under the plasma concentration versus time curve (AUC_0–48 h_) and mean residence time (MRT) was performed by means of a model independent method (noncompartmental) using Thermo Kinetica software (version 5.0.11, Philadelphia, PA). For both *in vitro* and *in vivo* studies, one-way ANOVA was used to compare the means of data followed by the Tukey Kramer Post-test using Graph Pad Instat® software (San Diego, CA). Differences were considered statistically significant at *p* ≤ 0.05.

## Results

### *In vitro* studies

#### PS and EE% of Alb MS and Enox-Alb MS

Size range around 10 μm were previously reported to be suitable for passive lung targeting following IV drug administration (Gupta & Hung, 1989; Pande et al., 1991; Kutscher et al., 2010). Many factors are known to influence the size and size distribution of Alb MS, such as Alb concentration, surfactant type and concentration, stabilization degree, etc. (Gupta & Hung, 1989; Urs et al., 2010). First, the process parameters producing MS of the desired target size were assessed.

**Figure 1.**
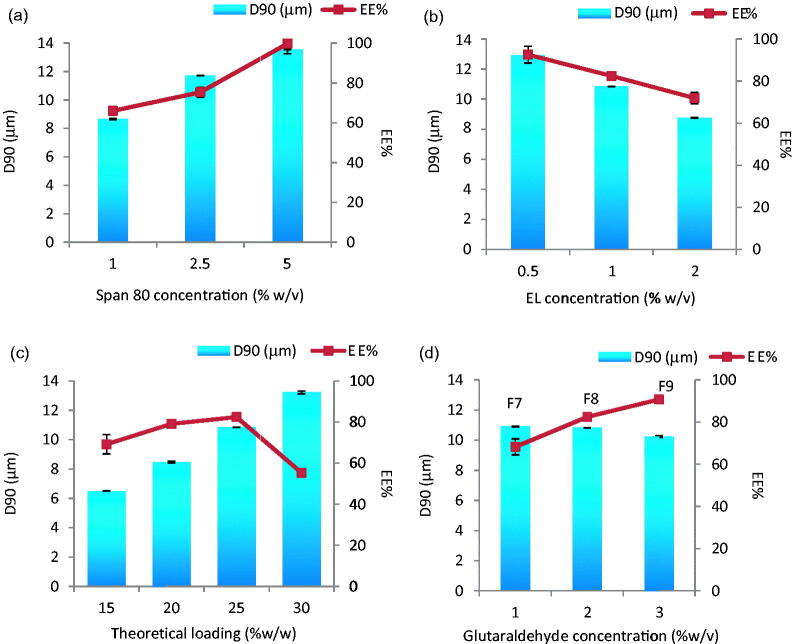
Effect of (a) span 80 concentration (% w/v), (b) EL concentration (% w/v), (c) theoretical loading (% w/w) and (d) glutaraldehyde concentration (% w/v) on the particle size and entrapment efficiency % w/w of Enox-Alb MS. D90 is the diameter where 90% of the population lies below this value.

Although phase volume ratio has always been known to be one of the most important factors affecting particulate size (El-Mahdy et al., 1998) it did not cause significant change in the PS in this study. Due to the expected high impact of surfactant type and concentrations for the production of particles with the desired size, we started with wide concentration ranges (1 and 10% w/v) for span 80 and (1 and 5% w/v) for lecithin. Both 10 and 5% concentrations yielded MS with sizes 12–15 μm and thus were eliminated from further experiments (results not shown). Smaller MS were only obtained with 1% of either span 80 or EL. MS size optimization process was then carried out by fine tuning of both surfactant concentrations: 1, 2.5 and 5 for span 80 and 0.5, 1 and 2% for EL. Using a phase volume ratio of 0.04, drug-loaded MS revealed gradual changes in both PS and EE% with surfactants concentrations rise (Figure 1a,b). A positive correlation was depicted in case of span 80 and a negative one with EL. The highest EE% of 99.70 ± 4.90 accompanied the largest (13.56 μm) sized particles when 5% span 80 was employed and 92.5%±4.90 in case of 12.91 μm ± 0.01 sized EL-stabilized MS (0.5%). The targeted MS size of 10.84 μm was generated only at 1% EL with an EE% of 82.42%. The effect of theoretical drug loading (15–30%) was studied with this formula where larger MS were obtained by increasing drug content (*p* < 0.0001) (Figure 1c). The targeted PS and the maximum EE% were only guaranteed at 25% drug loading. EE% enhancement and prevention of drug leakage during processing was possible by addition of the crosslinker glutaraldehyde. In fact, significant increase in EE% (*p* < 0.05) from 82.42 to 90.7% accompanied the crosslinker concentration increase to 3%. The presence of glutaraldehyde was quite effective for EE% enhancement with no effect on PS (F7, F8 and F9 in Figure 1d).

#### Enoxaparin release and particle behavior in PBS pH 7.4

**Figure 2.**
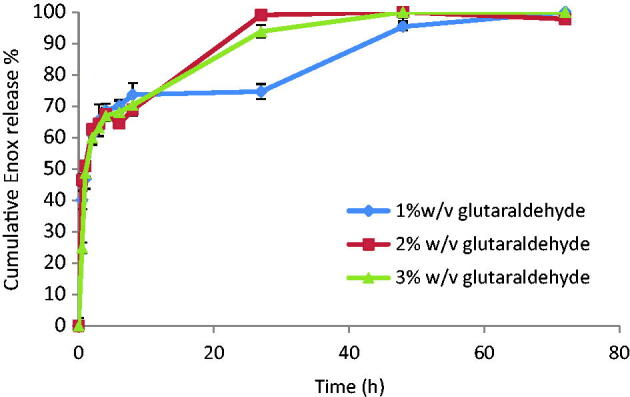
Release profiles from Enox-Alb MS in PBS pH 7.4 at 37 °C.

Although crosslinkers are successful in drug leakage prevention, yet they might negatively affect drug release. So, release from F7, F8 and F9 was studied. They all show a burst release during the first hour followed by a slower, sustained release of the remaining drug over the next 2–3 days depending on glutaraldehyde concentration (Figure 2). F8 and F9 released 100% of the drug in 48 h. Since F8 contained lower crosslinker concentration, it was scrutinized in the following *in vitro* and *in vivo* studies.

The microscopic images of fluorescent FITC-dextran-loaded Alb MS incubated in PBS pH 7.4 (Figure 1S) describes a slight swelling after 1 h which increases after 6 h and finally particle disintegration at 48 h.

#### Scanning electron microscopy

**Figure 3.**
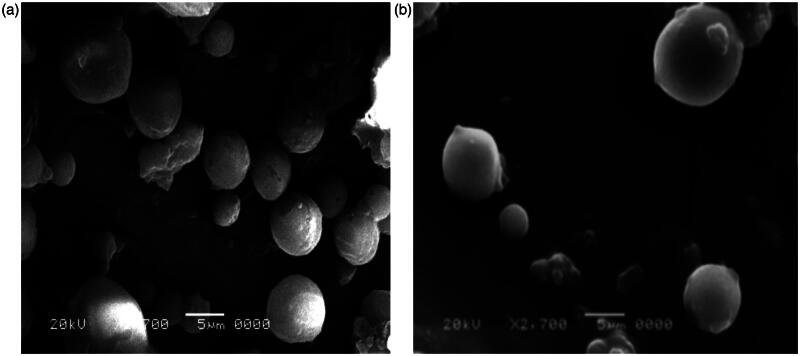
SEM micrographs of freeze dried (a) plain and (b) Enox-Alb MS of formula F8. Magnification ×2700.

Enox-Alb MS exhibited a slightly larger diameter and smoother surface compared to their drug-free counterparts (Figure 3a and b). The majority of the drug-loaded particles were in the target size range of 10 μm confirming the results obtained by laser diffraction analysis.

#### DSC and FT-IR spectroscopy

DSC thermograms of plain and drug-loaded MS are shown in Figure 2(S). Enox, like heparin, is composed of repeating disaccharide units of d-glucosamine and uronic acid linked by 1, 4 interglycosidic bonds. Its main functional groups are COO^−^, SO^3−^, OH, NH and C–O–C. The FT-IR spectrum of Enox shows two peaks at 3443.6 and 1623 cm^−1^ corresponding to O–H and COO^–^ stretching vibrations, respectively. Stretching vibrations of S = 0 sulfate ions and C–O–C ether groups can be seen at 1235.8 and 1044.4 cm^−1^, respectively (Yang et al., 2006; Bagre et al., 2013).

**Figure 4.**
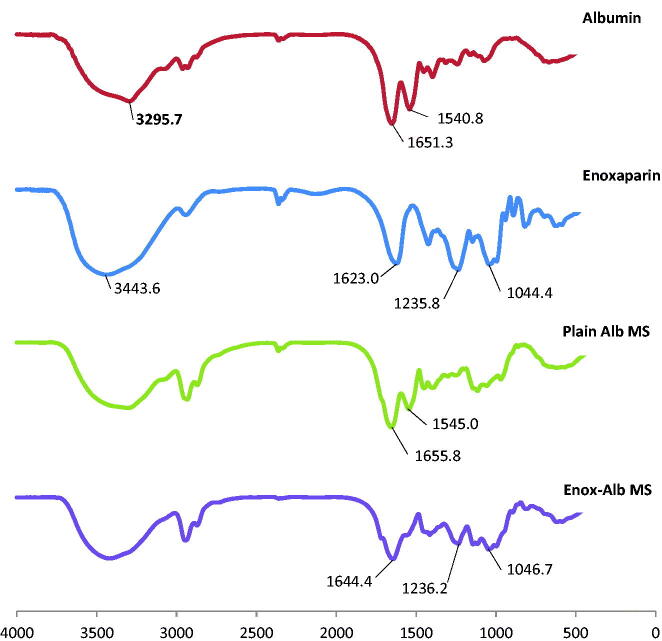
FT-IR spectra of albumin, enoxaparin, plain Alb MS and Enox-Alb MS.

The characteristic bands of both amide groups of Alb were present in plain Alb MS but a shift in 1651.3 cm^−1^ peak (corresponding to the C = 0 stretch in protein amide I) to 1644.4 cm^−1^ and loss of 1540.8 cm^−1^ peak (corresponding to amide II band) in loaded MS were noted. Moreover, loss of 1623 cm^−1^ peak (corresponding to COO^–^ stretching vibration) of Enox accompanied with a reduction in intensity of the 1235.8 cm^−1^ peak (corresponding to S = 0 sulfate ions vibration) was seen (Figure 4).

### *In vivo* studies

#### Lung localization and biodegradability

**Figure 5.**
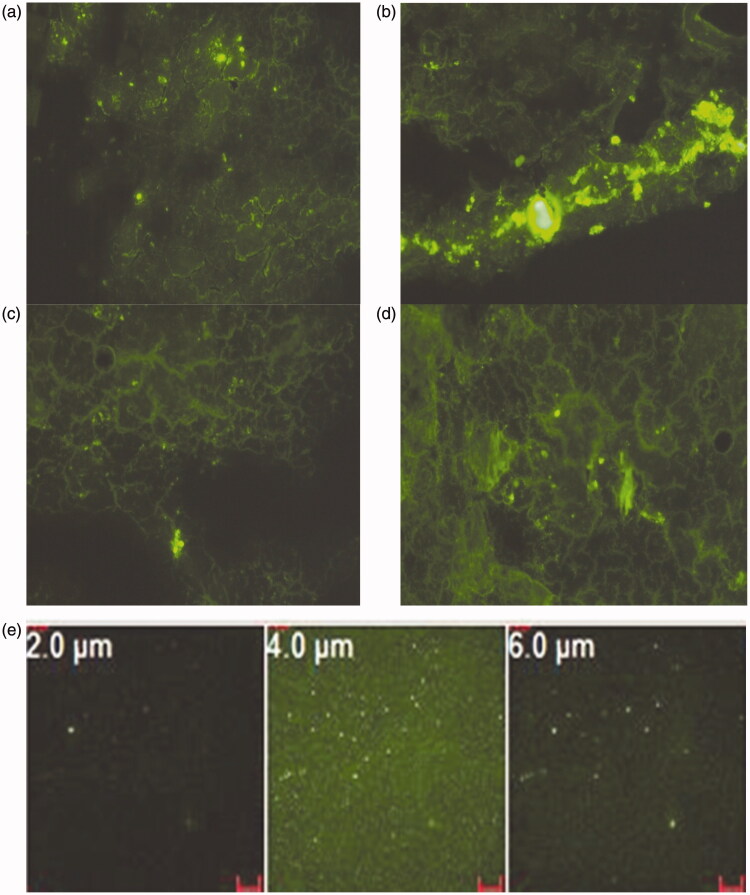
Fluorescent microscopic images of microtome sections of rat lung (a) 10 min, (b) 6 h, (c) 24 h, (d) 48 h post-IV administration of FITC-dextran loaded Alb MS and (e) confocal microscopic images of rat lung at various depths, 30 min post-IV administration of FITC-dextran loaded Alb MS. Magnification ×200.

The fluorescent microscopic images of microtome sections of rat lung at different time intervals following IV administration of FITC-dextran-labeled Alb MS are shown in Figure 5. Ten minutes following its bolus IV administration, the MS were rapidly distributed to the lungs as depicted from *in situ* tissue localization (Figure 5a). An intense fluorescence emitted from Alb MS is clearly distinguished from the faint and diffuse fluorescence of endogenous lung autofluorescence. Six hours post-drug administration, maximum particle swelling was detected, matching *in vitro* fluorescent microscopic images. Starting from the 24th hour, the fluorescent signal in the lung diminished. Although the particles were still present in the lung tissues 48 h post-injection, significant reductions in their numbers and sizes occurred ensuring biodegradation. The lung biodistribution pattern was also assessed on tissue cryosections using confocal images. A uniform and diffuse tissue distribution was seen 30 min following IV injection (Figure 5e). Although being uniformly disseminated at various depth (*Z* ranging from 2 to 6 μm), MS were mostly frequent at a *Z* value of 4 μm.

#### Histopathological study

**Figure 6.**
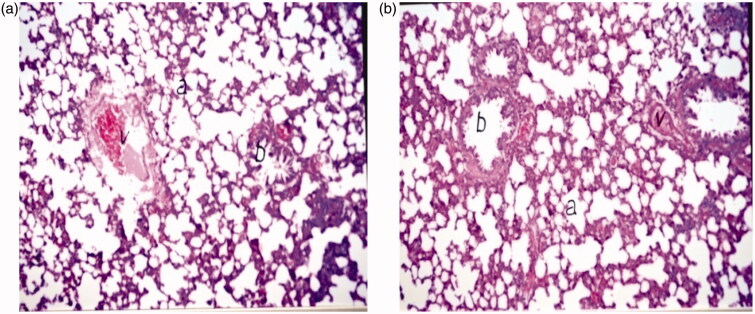
Light photomicrograph of rat lung 24 h post-IV administration of (a) D5W and (b) formula F8 at a magnification of ×160 (a: air alveoli, b: bronchiol and v: blood vessel).

Lungs, collected 24 h after the injection of control and Enox-Alb MS, were sectioned and stained with hematoxylin. Photomicrographs of different rat lung tissues sections following F8 injection revealed normal histological structures of the bronchioles, air alveoli and blood vessels in both groups with no histopathological alterations (Figure 6). No emboli or tissue infarction was observed macro- and/or microscopically in any of the samples.

#### Pharmacokinetic study

Previously published data had shown that a minimum plasma antifactorXa level of 0.1–0.2 IU/mL is required to produce an antithrombotic effect in rat model (Tang et al., 2010). In addition, an upper limit of 1.2 IU/mL has been recommended to avoid increased bleeding risk in humans at higher doses (Lyons et al., 2011).

**Figure 7.**
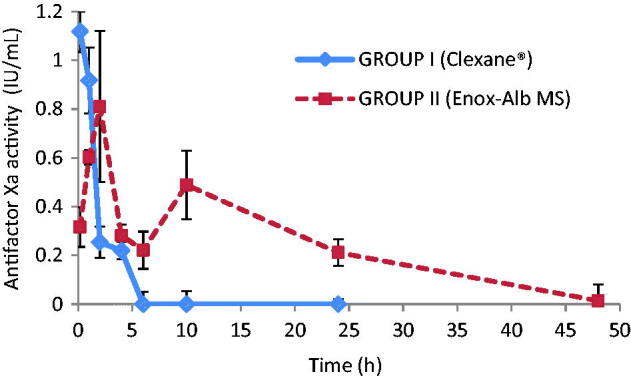
AntifactorXa activity versus time curve for Enox following IV administration of Clexane® and Enox-Alb MS (F8).

Drug concentration profile in whole blood following a single IV administration of Enox-Alb MS formulation is presented in Figure 7 and compared to the PK profile of the marketed product Clexane®.

Apparently, post-IV administration of Clexane®, antifactorXa activity reached a *C*_max_ of 1.12 ± 0.06 IU/mL. Interestingly, Enox-Alb MS exhibited an altered Enox plasma-antifactorXa activity-time profile manifested by the presence of two *C*_max_. One reaching 0.81 ± 0.22 IU/mL at 2 h post-injection, falling to 0.22 ± 0.04 IU/mL at 6 h and the second but lower *C*_max2_ reached 0.49 ± 0.09 IU/mL at 10 h followed by a decline to a subtherapeutic level (<0.1 IU/mL) at 48 h. It should be noted that neither of the two *C*_max_ exceeded the toxic drug level (>1.2 IU/mL). Furthermore, the long circulating Enox-Alb MS retained their antifactorXa activity for 38 h, while Clexane® had non-detectable activity after 6 h.

AUC_0–48_ of the prepared formulation was of 7.02 ± 1.39 IU h/mL, which was of a significantly higher value than that achieved by IV market product (2.26 ± 0.44 IU h/mL) (*p* < 0.05). The MRT of the former was consequently markedly enhanced (12.35 h ± 2.22) versus 3.01 h ± 1.11, in case of market product (*p* < 0.05).

## Discussion

Smaller size was set as the goal during process parameters setting with plain microspheres to account for possible PS increases following drug incorporation. Accordingly, the respective target sizes were 8 and 10 μm for plain and drug-loaded MS.

Impediment of the emulsification efficiency at a given stirring rate may occur due to insufficient amount of surfactant as in case of low lecithin concentration or due to viscosity increase by higher span 80 concentration delivering larger MS (Francis et al., 2011). The larger particles, obtained with both surfactants, have a smaller surface area to volume ratio and thus a lower potential for drug escape to the external phase (Li et al., 1999). This was consistent all over our study except at 1% concentration of both surfactants. Being a strongly hydrophilic drug, Enox was better encapsulated in presence of EL with the higher HLB (Bai & Ahsan, 2010) than with span 80 with a 4.6 HLB value (Sjöström et al., 1993). Besides, the higher EL concentration resulted in its accumulation at the droplet surface with a consequent improved protection against coalescence (Jeffery et al., 1993). The reverse occurred with span 80 where higher concentrations resulted in increased oil phase viscosity producing larger particles (Francis et al., 2011) and impeding drug escape to the aqueous phase (99.70 ± 4.90%). Optimum drug to polymer ratio was required to achieve maximum EE%, beyond which the polymer amount was probably insufficient to encapsulate the added drug load (Shailesh et al., 2010). Accordingly only 25% drug theoretical loading generated MS with the highest EE%. A commonly used crosslinker, glutaraldehyde, might increase drug EE with/without affecting the PS (Vural et al., 1990). Its long-term use has been associated with toxicity issues so it is important to use it at an optimal minimum concentration to avoid potential health problems (Gülsu et al., 2012).

Drugs can be associated with Alb MS by either adsorption onto the particle surface or inclusion within the matrix (Gupta et al., 1986). A burst initial phase in the release profiles of drugs encapsulated in Alb MS has been reported by various authors (Gupta et al., 1986; Vural et al., 1990; Mathew et al., 2007; Gülsu et al., 2012) and has been attributed to fast dissolution of loosely bound drug located near the surface followed by prolonged release of the remaining drug payload encapsulated deeper within the MP.

*In vitro* and *in vivo* images of fluorescent MS are highly correlated. After 60 min of incubation *in vitro*, MS are still intact with no signs of degradation supporting the hypothesis that the burst release was due to the surface-associated drug rather than the encapsulated payload. Five hours later, the swollen particles lost their smooth surface, starting degradation while delivering slowly their payload (Das et al., 2011). Both DSC and FT-IR studies indicated drug encapsulation with possible polymer–drug interaction. *In vivo* studies also proved direct lung targeting 10 min following IV injection, and swelling was obvious after 6 h. MS degradation started after 24 h and most MS have degraded leaving only a few of them intact by the 48th hour. The slow and prolonged clearance of MS from the lung tissues would be useful for local prophylaxis and treatment of PE. Pulmonary vasculature exposed to Enox MS for longer durations will benefit from a sustained anticoagulant activity. Confocal microscopy proved the random distribution of Enox-Alb MS within the lung.

Multiple peaking in plasma concentration–time curves of drugs is a phenomenon occasionally encountered in PK. It can occur as a consequence of a number of different mechanisms including sustained formulation. The preparation was apparently divided into fast- and slow-release fraction components, each fraction releasing the drug at a different rate constant (Davies et al., 2010). The weakly adsorbed surface drug was at the base of initial *C*_max1_ and the fraction embedded in the matrix needing particle disintegration corresponded to the second maximum concentration. These findings were also confirmed by both *in vitro* and *in vivo* fluorescence images.

A close relationship exists between the circulating time and clearance pharmacokinetic profile on one hand, and the behavior of carriers on the other hand (Wang et al., 2012). The increase in AUC of Alb MS compared to market product can be explained by a dual mechanism; first, prolongation of the drug’s anticoagulant activity by MS deposition in the lung capillary beds, protecting it from body elimination mechanisms, secondly, as low drug liberation from the prepared Alb MS matrix. Moreover, the short MRT (∼3 h) obtained for Enox market product (Clexane®), could be attributed to its hydrophilic nature, typically, undergoing rapid renal clearance with poor reabsorption after glomerular filtration (Kadam et al., 2012).

## Conclusion

The present study proved, for the first time, that compared to free injected drug, passively lung targeted Enox-Alb MS had an effective sustained antithrombotic activity in rats. Rapid and prolonged activity with an even lung tissue distribution paved the way for injectable MS of Enox for prophylaxis and treatment of PE, one of the most common causes of death.

## Supplementary Material

suppl._material_1.docx

## Notes

**Supplementary material available online**
