# Supplementary material for: Passive targeting and lung tolerability of enoxaparin microspheres for a sustained antithrombotic activity in rats
Source: Drug Deliv. 2017 Feb 3;24(1):243–51. doi: 10.1080/10717544.2016.1245368 (PMC8241188; doi:10.1080/10717544.2016.1245368)
Supplement: suppl._material_1.docx [file IDRD_A_1245368_SM3142.docx]

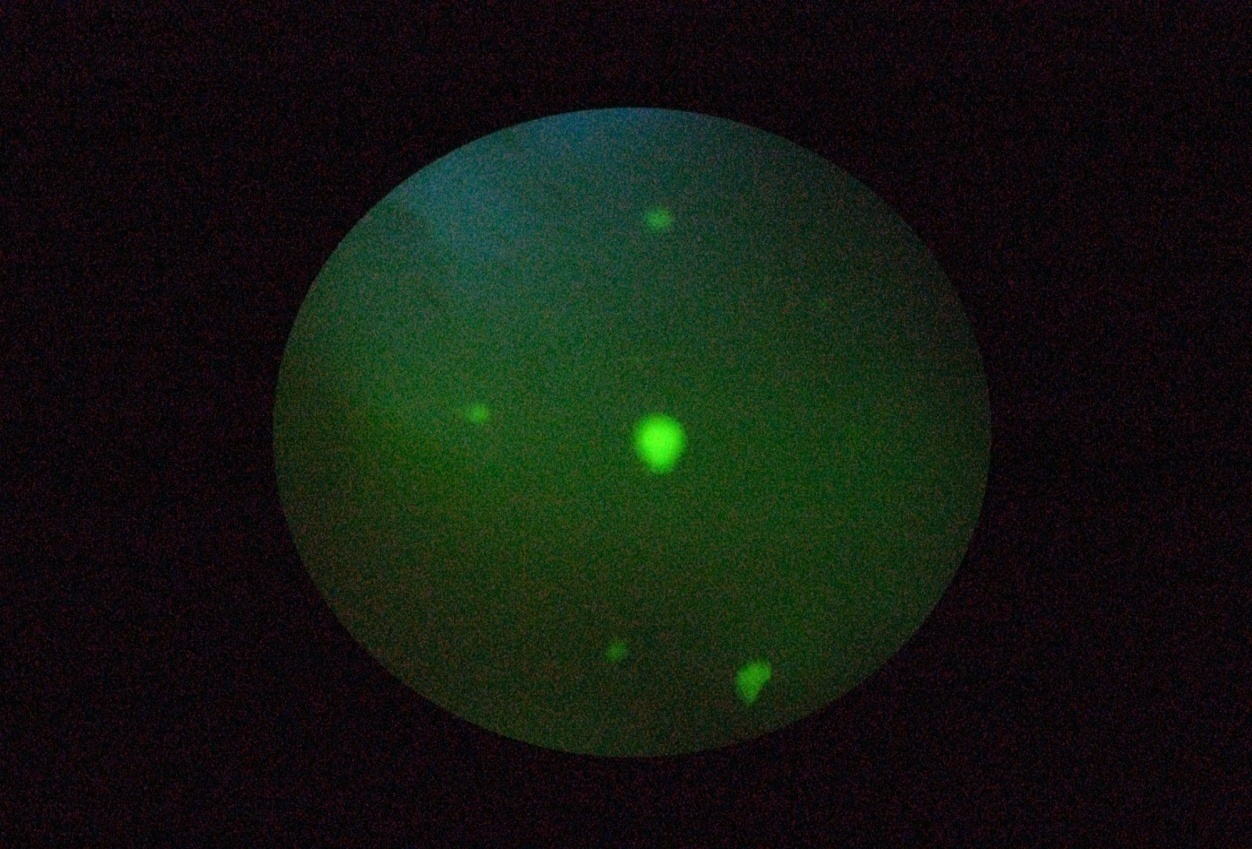

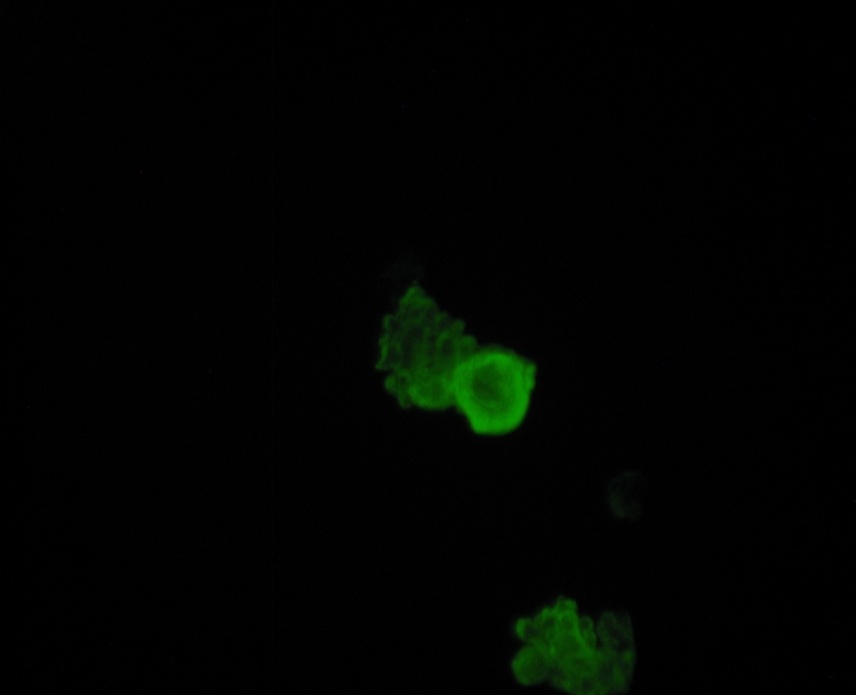

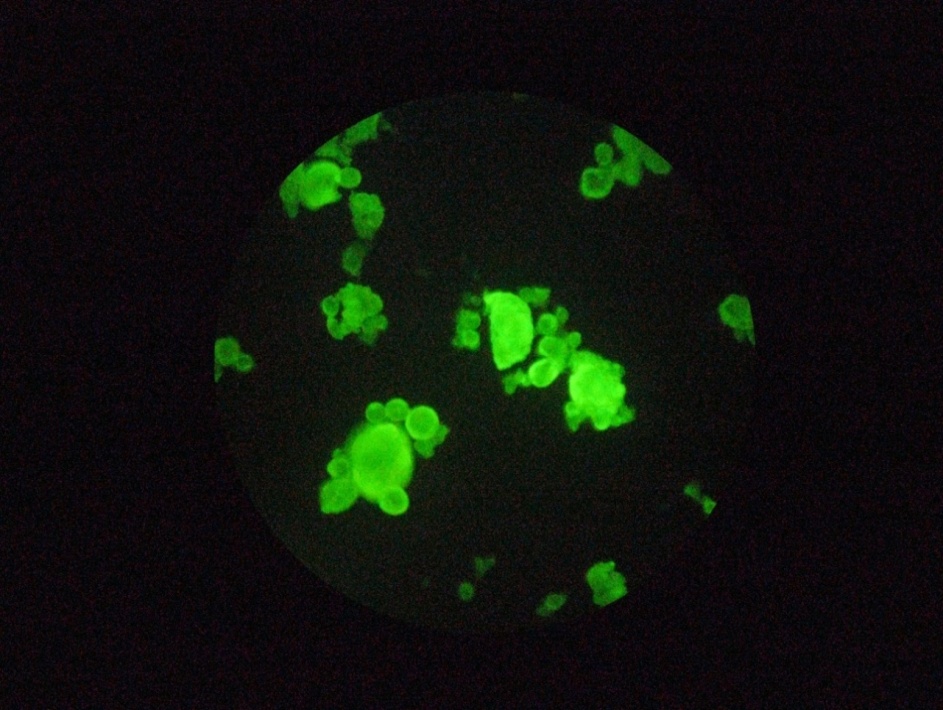


**(b)**

**(a)**

**(c)**

**Figure 1S. Microscopic images of FITC-dextran-loaded Alb MSincubated in PBS pH 7.4 for a) 60 minutes, b) 6 hours and c) 48 hours. Magnification x400.**

Exotherm

Endotherm

**Figure 2S. Differential scanning calorimetry chromatograms of Enoxaparin, albumin, plain Alb MS and Enox-Alb MS.**


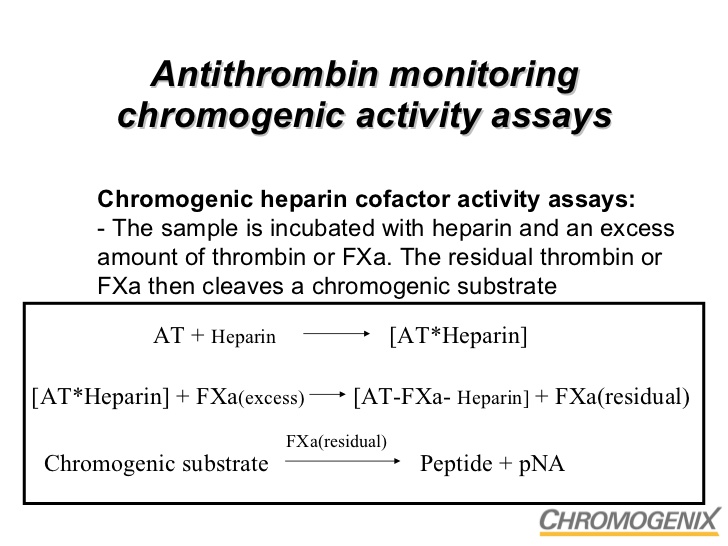


(Yellow color)

Chromogenic peptide substrate

**Figure IS. Schematic diagram of principle of LMWH chromogenic assay (Kinetichrome^TM^ Heparin brochure)**
